# A Dataset of Deep-Sea Fishes Surveyed by Research Vessels in the Waters around Taiwan

**DOI:** 10.3897/zookeys.466.8523

**Published:** 2014-12-19

**Authors:** Kwang-Tsao Shao, Jack Lin, Hsin-Ming Yeh, Mao-Yin Lee, Lee-Sea Chen, Hen-Wei Lin

**Affiliations:** 1 Biodiversity Research Center, Academia Sinica, No. 128, Sec. 2, Academia Rd. Nankang Dist., 115, Taipei, TAIWAN; 2 Coastal and Offshore Resources Research Center, Fisheries Research Institute, COA, No.6, Yugang N. 3rd Rd., Qianzhen Dist., 806, Kaohsiung City, TAIWAN

**Keywords:** Deep-Sea Fish Fauna, Otter Trawl, Beam Trawl, IKMT, Catalog of Life, Barcode of Life, Encyclopedia of Life

## Abstract

The study of deep-sea fish fauna is hampered by a lack of data due to the difficulty and high cost incurred in its surveys and collections. Taiwan is situated along the edge of the Eurasia fig, at the junction of three Large Marine Ecosystems or Ecoregions of the East China Sea, South China Sea and the Philippines. As nearly two-thirds of its surrounding marine ecosystems are deep-sea environments, Taiwan is expected to hold a rich diversity of deep-sea fish. However, in the past, no research vessels were employed to collect fish data on site. Only specimens, caught by bottom trawl fishing in the waters hundreds of meters deep and missing precise locality information, were collected from Dasi and Donggang fishing harbors. Began in 2001, with the support of National Science Council, research vessels were made available to take on the task of systematically collecting deep-sea fish specimens and occurrence records in the waters surrounding Taiwan. By the end of 2006, a total of 3,653 specimens, belonging to 26 orders, 88 families, 198 genera and 366 species, were collected in addition to data such as sampling site geographical coordinates and water depth, and fish body length and weight. The information, all accessible from the “Database of Taiwan’s Deep-Sea Fauna and Its Distribution (http://deepsea.biodiv.tw/)” as part of the “Fish Database of Taiwan,” can benefit the study of temporal and spatial changes in distribution and abundance of fish fauna in the context of global deep-sea biodiversity.

## Data resources

The data underpinning the analyses reported in this paper are deposited in the GBIF, the Global Biodiversity Information Facility, http://taibif.tw/ipt/resource.do?r=deep-sea-fishes.

## Taxonomic coverage

“ Fishes of the World” (Nelson 2006) was used as a taxonomic reference for this work.

**Figure 1.**
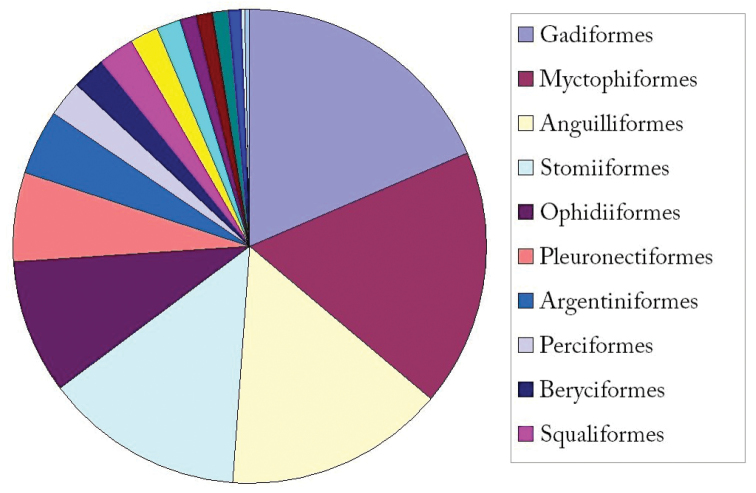
Taxonomic coverage (by order).

**Figure 2.**
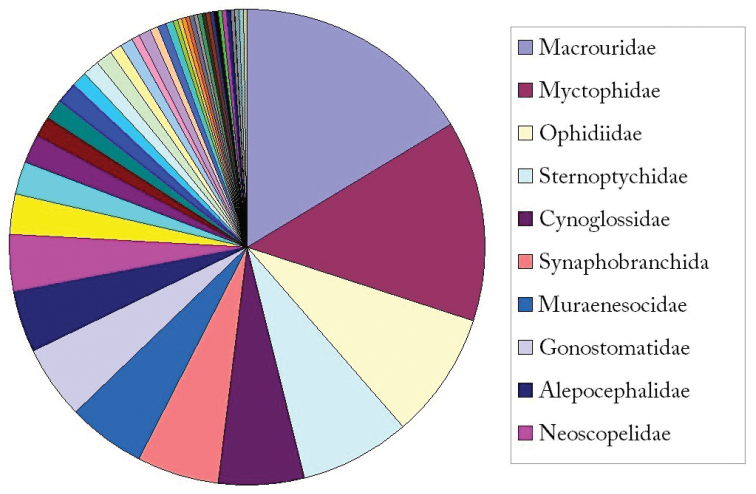
Taxonomic coverage (by family).

**General taxonomic coverage description:** The coverage of this dataset includes Class Actinopterygii (3,496/3,653), Class Chondrichthyes (156/3,653) and Class Myxini (1/3,653). The top 10 orders are Gadiformes, Myctophiformes, Anguilliformes, Stomiiformes, Ophidiiformes, Pleuronectiformes, Argentiniformes, Perciformes, Beryciformes and Squaliformes (Figure 1). The top 10 families are Macrouridae, Myctophidae, Ophidiidae, Sternoptychidae, Cynoglossidae, Synaphobranchidae, Muraenesocidae, Gonostomatidae, Alepocephalidae and Neoscopelidae (Figure 2).

## Taxonomic ranks

**Kingdom:** Animalia

**Phylum:** Chordata

**Class:** Actinopterygii, Chondrichthyes, Myxini

**Order:** Albuliformes, Anguilliformes, Argentiniformes, Aulopiformes, Beryciformes, Carcharhiniformes, Chimaeriformes, Gadiformes, Gasterosteiformes, Gonorhynchiformes, Lamniformes, Lophiiformes, Myctophiformes, Myliobatiformes, Myxiniformes, Ophidiiformes, Perciformes, Pleuronectiformes, Rajiformes, Saccopharyngiformes, Scorpaeniformes, Squaliformes, Stephanoberyciformes, Stomiiformes, Tetraodontiformes, Torpediniformes

**Family:** Acropomatidae, Alepocephalidae, Aphyonidae, Aploactinidae, Bathyclupeidae, Bathylaconidae, Bothidae, Bramidae, Bregmacerotidae, Bythitidae, Callionymidae, Caristiidae, Centrophoridae, Cepolidae, Ceratiidae, Champsodontidae, Chaunacidae, Chiasmodontidae, Chimaeridae, Chlorophthalmidae, Colocongridae, Congridae, Cottidae, Cynoglossidae, Dalatiidae, Diretmidae, Ereuniidae, Etmopteridae, Eurypharyngidae, Gempylidae, Gonorynchidae, Gonostomatidae, Halosauridae, Himantolophidae, Hoplichthyidae, Ipnopidae, Linophrynidae, Lophiidae, Macrouridae, Melamphaidae, Melanocetidae, Melanonidae, Microstomatidae, Moridae, Muraenesocidae, Muraenidae, Myctophidae, Myxinidae, Nemichthyidae, Neoscopelidae, Nettastomatidae, Nomeidae, Notacanthidae, Ogcocephalidae, Oneirodidae, Ophichthidae, Ophidiidae, Ostracoberycidae, Paralepididae, Paralichthyidae, Percichthyidae, Percophidae, Peristediidae, Phosichthyidae, Poecilopsettidae, Priacanthidae, Pseudocarchariidae, Rajidae, Rondeletiidae, Scopelarchidae, Scorpaenidae, Scyliorhinidae, Serrivomeridae, Sparidae, Squalidae, Sternoptychidae, Stomiidae, Synaphobranchidae, Syngnathidae, Synodontidae, Tetraodontidae, Torpedinidae, Trachichthyidae, Triacanthodidae, Trichiuridae, Triglidae, Urolophidae, Zoarcidae

## Spatial coverage

**Figure 3.**
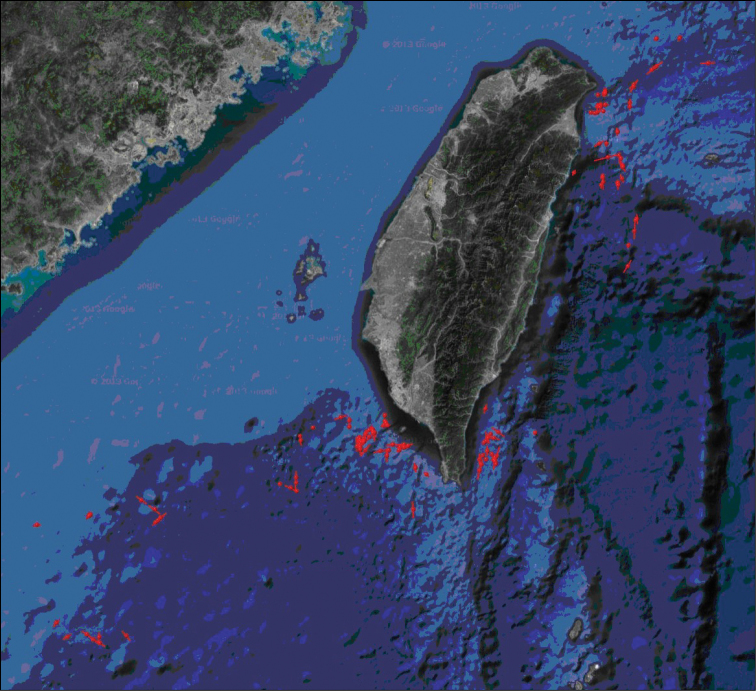
Spatial coverage (sampling routes).

**General spatial coverage:** Seas around Taiwan (Figure 3)

**Coordinates:** 20°38'52.8"N and 25°23'2.4"N Latitude; 117°17'42"E and 123°0'43.2"E Longitude

**Temporal coverage:** May 20, 2001–August 27, 2006

## Methods

**Study extent description:** The surveys were carried out from 2001 to 2006 in waters off northeastern Taiwan (Okinawa Trough), eastern Taiwan, southeastern Taiwan (Western Pacific) and southwestern Taiwan (South China Sea).

**Sampling description:** The research vessels used were “R/V Fishery Researcher I,” “R/V Ocean Researcher I” and “R/V Ocean researcher III.” Constrained by limited cable length, the maximum depth sampled was 4,460 meters. Major equipment used were otter trawl, French type beam trawl of 4 m span, ORE type beam trawl of 3 m span and Isaacs-Kidd midwater trawl (IKMT). Once the nets reached the sea bottom, they were towed for one hour at a ground speed of 1.5–2.5 knot for otter trawls and 1.0–1.5 knot for others.

**Quality control description:** All the scientific names of fish samples were validated by the updated fish checklist in the “Fish Database of Taiwan” or TaiCOL (http://col.taibif.tw; formerly TaiBNET, http://taibnet.sinica.edu.tw) before they were entered into database. Afterward, they were validated again by matching against FishBase and Catalog of Fishes, California Academy of Sciences for further correction. If a specimen was rare or it might belong to an undescribed or new species, it was photographed in fresh and then both the specimen and its tissue sample were catalogued and deposited at the Biodiversity Research Museum of Biodiversity Research Center, Academia Sinica (ASIZP of BRCAS). The latitude and longitude of trawling routes were plotted on Google Maps and outlier detection was conducted.

### Step description:

Step1: Sampling locality and water depth were recorded.

Step2: Specimens were roughly classified and counted either right on board or when they reached the shore.

Step3: Specimens were shipped back to the lab for species identification, body length and weight measurement, and picture taking.

Step4: Specimens were fixed in 10% Neutral Buffered Formalin for one month. Next, they were cleaned with water and preserved in 70% alcohol.

## Project details

**Project title:** Survey of Deep-Sea Fish Diversity by Research Vessels in Taiwan Waters.

**Personnel:** Kwang-Tsao Shao (Project Director), Jack Lin (Software Engineer and Database Manager), Hsin-Ming Yeh, Mao-Yin Lee, Hsuan-Ching Ho, Yun-Chih Liao, Hen-Wei Lin (field work, fish identification, data collection and analysis).

**Funding:** Ministry of Science and Technology (previously National Science Council), Executive Yuan, R.O.C. (Taiwan).

**Study area descriptions/descriptor:** Taiwan is located on the eastern edge of Asian continental shelf. To the west of Taiwan is the shallow Taiwan Strait, to the northeast is the Okinawa Trough (maximum depth 2,716 m), to the east is the complex and diverse Philippine Sea (with deep oceanic trenches), and to the south is the South China Sea (maximum depth 5,016 m). These deep-water environments were where the surveys were carried out.

**Design description:** This study focused on Taiwan’s deep-sea fish fauna, which so far hasn’t been investigated much, and hoped to learn if the fauna varies depending on the sea area, current and water depth. All the specimens caught went through taxonomic identification and had their collection time, water depth and coordinates recorded. A geographic information system (GIS) on their distributions was established in order to provide references for future academic researches as well as resource development, management and assessment. One or several specimens per fish species were selected to have their photos taken in color. Keeping to the Barcode of Life tissue preservation techniques, a small piece of tissue was excised, preserved in 90–95% alcohol and stored at BRCAS in liquid nitrogen canisters. Backup tissue samples were also stored at Livestock Research Institute, COA to facilitate the study of molecular biology and genetics later. The voucher specimens and whole fish specimens were deposited at BRCAS. The specimen information was entered into the Fish Database of Taiwan and is freely accessible to all.

## Datasets

**Dataset description:** The dataset includes station number, locality name, water depth, collection date, latitude, longitude, family name, species name and Chinese common name. Since the number of individuals and the weight and length of each specimen are also included, the data can be used to calculate biodiversity indices, K-dominance (A-B-C) curve and community structure analysis by applying various clustering or ordination methods. They can form a good baseline for the time period of 2001-2006. If more data can be collected in the future, comparisons can be performed and the question of whether deep-sea fish diversity is declining under anthropogenic and climate change impacts can be assessed. The collected specimens (voucher and tissue sample) deposited at BRCAS are open to all users for taxonomic and ecological researches so that studies on new species, phylogeny and zoogeography of deep-sea fishes can be published. Some images and morphological data can also be used by the global databases of FishBase, OBIS, GBIF and EOL. Additionally, detailed analyses on the body size of certain species can generate valuable information on the fish’s early life history and its inshore or offshore migration and recruitment.

**Object name:** Darwin Core Archive A Dataset of Deep-Sea Fishes Surveyed in the Waters around Taiwan

**Character encoding:** UTF-8

**Format name:** Darwin Core Archive format

**Format version:** 1.0

**Distribution:** http://taibif.tw/ipt/archive.do?r=deep-sea-fishes

**Publication date of data:** 2014-08-26

**Language:** English

**Metadata language:** English

**Date of metadata creation:** 2014-08-26

**Hierarchy level:** Dataset
